# Inhibition of Growth and Induction of Apoptosis of Human Prostate Cancer Cells by Enzymatic Blockage of Kallikreins

**DOI:** 10.1155/proc/7871208

**Published:** 2026-01-11

**Authors:** Fabienne Lehner, Souzan Salemi, Christopher Millan, Christoph Kündig, Daniel Eberli

**Affiliations:** ^1^ Department of Urology, University Hospital Zurich, 8091, Zurich, Switzerland, usz.ch; ^2^ Laboratory for Urologic Oncology and Stem Cell Therapy, Department of Urology, University Hospital Zurich, 8952, Schlieren, Switzerland, usz.ch; ^3^ Med Discovery SA, 1066, Epalinges, Switzerland

**Keywords:** androgen receptor, apoptosis, kallikrein 2, prostate-specific membrane antigen, theranostics

## Abstract

**Background:**

Most therapy options for castration‐resistant prostate cancer (CRPCa) target the androgen axis. Human kallikrein–related peptidase (KLK) 2, a serine protease, is a downstream target gene of the androgen receptor (AR) involved in cancer progression, but also known to have an AR‐independent function. Tissue KLKs, especially KLK2, are promising targets for therapy in advanced PCa because of their high PCa specificity and their correlation to the rising cancer grade and stage. By inhibition with the recombinant protease inhibitor MDPK67b targeting KLK2 and other trypsin‐like KLKs including KLK4 and KLK14, we investigated the antitumor response and the influence on AR downstream target genes with MDPK67b in PCa cell lines in vitro.

**Methods:**

Human PCa cells were cultured in a charcoal‐stripped media and treated with MDPK67b (0.75 mg/mL). Cell viability was measured by CellTiter‐Glo luminescent assay, cell death by flow cytometry. Gene analysis of AR, PSA, and PSMA was performed by qPCR. Correlating protein levels were evaluated by immunoblotting and confirmed by immunocytochemical staining.

**Results:**

Treatment with 0.75 mg/mL MDPK67b led to a reduction of cell proliferation of 40% by day 5 in androgen‐sensitive LNCaP cells. Immunostaining confirmed the decrease in cell proliferation by antibody labeling of Ki‐67. Treatment induced apoptosis, which was visible by flow cytometry of annexin V in LNCaP cells. Further, MDPK67b induced a reduction in AR and PSA gene and protein expression but upregulated PSMA, a target for PCa imaging and therapy.

**Conclusion:**

Treatment with MDPK67b demonstrates a significant antitumor effect by relevant reduction in cell proliferation and upregulation of apoptosis in LNCaP cells. Blockage of secreted KLKs can downregulate the AR and thereby influence its downstream target genes like PSA and PSMA. Upregulation of PSMA can lead to a theranostic, that is, therapeutic and diagnostic, advantage in clinics in a CR setting. Therefore, inhibition of KLKs represents a promising and AR‐independent approach to treat advanced and CRPCa.

**Trial Registration:**

ClinicalTrials.gov ID: NCT04644770

## 1. Introduction

Targeting the androgen receptor (AR) signaling axis is the main therapeutic focus in the management of advanced prostate cancer (PCa) [1]. Men progress rapidly into a castration‐resistant state upon initiation of androgen‐deprivation therapy (ADT) [2], which leads to a subsequent uncontrolled disease progression [3]. Therefore, AR‐independent treatment options are needed. Recent studies have revealed the importance of human kallikreins (KLKs) and especially human kallikrein 2 (KLK2) as a diagnostic marker and therapeutic target in PCa [4, 5].

To date, 15 KLKs that are homologous secreted serine proteases with trypsin‐ or chymotrypsin‐like activities with tissue‐specific expressions have been discovered [6]. KLK3, mainly known as prostate‐specific antigen (PSA), is the foremost clinically accepted serum tumor marker to diagnose and monitor PCa. Other KLKs have emerged as key players in PCa pathogenesis, particularly KLK2, KLK4, and KLK14, which are implicated in cancer progression through protease activity, AR modulation, and interaction with extracellular matrix components [7]. Especially KLK2 has become of great interest. It is sparsely produced in other tissues, but its expression is the highest in PCa tissue, and therefore specific [8]. Patient’s tissue samples revealed a rise of the KLK2 expression rate corresponding to disease severity, and therefore was highest in metastatic PCa specimens [9, 10]. Once secreted, KLK2 is able to perform auto‐activation as well as activation of KLK3/PSA giving it a distinct function, even though the two KLKs show an 80% gene homology [11]. KLK2’s protease potency is superior to that of PSA [12] and includes proteolysis of urokinase‐type plasminogen activator (uPA), protease‐activated receptors (PAR) 1&2 as well as degradation of insulin‐like growth factor binding proteins (IGFBP). The aforementioned factors are involved in extracellular matrix degradation and cancer progression [13, 14]. KLK2 influences cell growth as a downstream target gene of the AR, but it has been shown to have AR‐independent properties whereby it is able to modulate the AR through the coregulator ARA70 [15].

KLK4 and KLK14 display a broader tissue specificity. KLK4 has been implicated in promoting PCa progression by stimulation of tumor growth and metastasis through induction of cancer‐associated fibroblast phenotypes in prostate stromal cells [16], epithelial to mesenchymal transition‐like characteristics [17], and activation of matrix metalloproteinase‐1 (MMP‐1) [18]. Moreover, KLK4‐transfected PC3 cells exhibit greater attachment to the bone‐matrix proteins [19]. KLK14 demonstrated comparable in vitro activities as KLK2 or KLK4 as a PAR‐2 activator, yet has mainly been studied as a biological marker linked to advanced PCa [20].

Different mechanisms in the acquisition of castration resistance are described, including AR mutations, AR splice variants, bypass signaling, and intratumoral androgen synthesis [21]. Emphasizing the characteristics of KLKs, they represent a promising new pharmacological target in treatment of advanced and CRPCa. Cloutier et al. developed highly specific and reactive serpin toward KLK2, KLK4, and KLK14 by alteration of the reactive site loop of ***α*** 1‐antichymotrypsin using phage‐display technology. MDPK67b, a recombinant protease inhibitor targeting human kallikrein‐related peptidase 2, 4, and 14, covalently binds the KLKs extracellularly and irreversibly inactivates their catalytic abilities [22].

We hypothesize that the pharmacological inhibition of secreted KLKs, particularly KLK2, using the recombinant protease inhibitor MDPK67b, exerts an antitumor effect in PCa tumor cells in vitro by reducing proliferation and inducing apoptosis independently of the AR signaling and further modulates AR downstream targets such as PSA and PSMA providing a potential theranostic advantage in PCa.

## 2. Materials and Methods

### 2.1. Cell Lines and Cell Culture

LNCaP cells (ATCC CRL‐1740, Manassas, VA, USA) were cultured in RPMI 1640 medium with phenol red (Life Technologies, Thermo Fisher Scientific, Waltham, MA, USA). C4‐2 cells (ATCC CRL‐3314, Manassas, VA, USA) were maintained in T‐medium (supplemented with 10% fetal bovine serum [FBS; Gibco], 1% penicillin–streptomycin [100 U/mL and 100 μg/mL, respectively], insulin [5 μg/mL], triiodothyronine [T_3_; 13.6 pg/mL], apo‐transferrin [5 μg/mL], biotin [0.25 μg/mL], and adenine [25 μg/mL]; Life Technologies, Thermo Fisher Scientific) [23, 24]. DU145 cells were supplied by Dermadis SA (France) and maintained in high‐glucose DMEM/F‐12 medium (Life Technologies, ThermoFisher Scientific, Waltham, MA). Culture media were supplemented with 10% FBS and 1% penicillin/streptomycin. Cells were incubated at 37°C with 5% CO_2_, and culture medium was changed twice a week.

### 2.2. In Vitro Protease Inhibition Assays

Proteases were incubated with 20× molar excess of MDPK67b, for 60 min at 37°C. The reaction buffer consisted of 50 mM Tris pH 7.5 containing 150 mM NaCl, 0.1% BSA, and 0.01% Triton X‐100, except for testing of KLK7 (50 mM Tris‐HCl buffer pH 8.5 containing 150 mM NaCl, 50 mM EDTA, 0.1% BSA) and KLK3 (50 mM Tris‐HCl buffer pH 7.5 containing 150 mM NaCl, 0.01% Triton X‐100, 0.1% BSA, and 1 M sodium citrate). Concentrations of proteases required for testing (see Table 1 and Table 2) were dependent on their respective in vitro activities and calculated according to the supplier’s recommendation.

**Table 1:** KLK inhibitory profile of MDPK67b.

Protease	% inhibition by 20× molar excess of MDPK67b
KLK1	0
KLK2	100
KLK3	50
KLK4	100
KLK5	100
KLK6	68
KLK7	81
KLK8	82
KLK13	37
KLK14	100

*Note:* KLK proteases and inhibitor were incubated at a molar ration of 20:1. Residual protease activity was measured through addition of fluorescent peptide substrates to the preincubated protease–inhibitor complex. The percentage of KLK inhibition was calculated to 100 × [1 − (velocity in the presence of inhibitor/velocity of uninhibited control)].

**Table 2:** Stoichiometry of inhibition (SI) values of MDPK67b for selected Kallikrein‐related peptidases.

Protease	SI value
KLK2	2.1
KLK4	1.7
KLK5	5.1
KLK14	1

*Note:* SI values were calculated for KLKs which were fully complexed and inactivated by a 20× molar excess of MDPK67b.

Residual protease activities were detected by the addition of 20 μM fluorescent substrates, and fluorescence was measured with excitation at 340 nm (±15) and emission at 485 nm (±10) in black 96‐well plates using an FLx800 fluorescence microplate reader (Bio‐Tek Instruments, Inc., USA). Percentage of inhibition was calculated as 100 ∗ [1 − (velocity in the presence of inhibitor/velocity of uninhibited control)].

Stoichiometry of inhibition (SI) values were determined only for proteases which were completely inhibited by the addition of a 20× molar excess of MDPK67b. Proteases were incubated with varying concentrations of MDPK67b. After 1 hour incubation at 37°C in reaction buffer, the residual protease activities were detected by the addition of fluorescent substrate. The reported SI value corresponds to the abscissa intercept of the linear regression analysis of fractional velocity (velocity of inhibited enzyme reaction (vi)/velocity of uninhibited enzyme reaction (v0)) versus the molar ratio of the inhibitor to enzyme ([I0]/[E0]). This value corresponds to the molar excess of inhibitor molecules, which is required under test conditions for complete inhibition of the protease.

### 2.3. Pharmacological Compound

Cell lines were treated with a recombinant protease inhibitor targeting human kallikrein‐related peptidase 2, MDPK67b (a ACT6.7 variant without His‐tag; Med Discovery, Epalinges, Switzerland) [22], for up to 5 days. For all experiments, if not otherwise mentioned, three biological replicates were performed. Different concentrations were tested, and the most effective was used for further experiments. The vehicle control was composed of a buffer including Tris‐HCl 50 nM, NaCl 50 nM, and polysorbate 80 (Tween80) 0.05%, pH 8.5 (supplied by Dermadis SA, France). The media containing the compound was supplemented with 5% heat‐inactivated charcoal‐stripped FBS (F6765, Sigma‐Aldrich, Buchs, Switzerland) to avoid interference of growth with a possible androgen stimulation or any other possible unspecific binding of the compound. Heat inactivation was performed at 65°C for 30 min. The medium containing the compound was changed every alternate day. All experiments were performed over 5 days and in triplicate. Before treatment, the cells were starved overnight.

### 2.4. Cell Proliferation Assay

Cells were cultured in 96‐well plates (Corning Costar, Cat. No. 3997, NY, USA) at densities of 7 × 10^3^ cells (LNCaP), 4 × 10^3^ cells (C4‐2), and 2.5 × 10^3^ cells (DU145) per well until adherent. After overnight starvation with serum‐reduced media, different concentrations of MDPK67b were tested (0.1 mg/mL, 0.5 mg/mL, 0.75 mg/mL, 1 mg/mL). CellTiter‐Glo3D luminescent cell viability assay (Promega, Switzerland) was performed according to the protocol at days 1, 3, 5, and 7 after initial treatment. Briefly, 50 μL of CellTiter‐Glo 3D reagent and an equal amount of cell culture medium were added to each well. The contents were mixed for 2 min on an orbital shaker, and the plate was left at room temperature for another 20 min. Afterward, 90 μL of each well was transferred to a new white‐bottom 96‐well plate and luminescence was measured by a Cytation 5 Cell Imaging Multi‐Mode Reader (BioTek Instruments, VT, USA).

### 2.5. Flow Cytometry Analysis

For flow cytometry analysis, cells were treated with 0.75 mg/mL of MDPK67b for 5 days in a 25 cm^2^ flask (TPP, Trasadingen, Switzerland) at densities of approximately 14′000 for LNCaP, 8000 for C4‐2, and 4000 cells/cm^2^ for DU145. The cells were washed with PBS, trypsinized, and pelleted by centrifugation at 1400 rpm for 5 min at 4°C. For the detection of apoptosis, necrosis cells were directly labeled with FITC Annexin V (Kit 556,547, BD Pharmingen) and propidium iodide (PI) (100 μg/mL, P4170, Sigma‐Aldrich) according to the manufacturer’s instructions. After washing the cells with cold PBS twice, the pellet was resuspended in 350 μL of binding buffer. FITC Annexin V (5 μL) was added, and the resuspension was incubated in the dark at room temperature for 15 min. Immediately before measuring cell fluorescence using a Becton Dickinson FACS Canto flow cytometer, 5 μL of PI was added. Data were analyzed by FlowJo software v. 10.0.8 (Tree Star, Inc., Ashland, Oregon). Percentages of positive apoptotic and necrotic cells were compared to vehicle control.

### 2.6. Immunocytochemistry

All cancer cell types were seeded and treated as mentioned above in 4‐well chamber slides (LabTek, ThermoFisher Scientific, Switzerland). After fixation with 4% paraformaldehyde for 20 min, the cells were washed with PBS, permeabilized for 5 min with 0.5% Triton X‐100 in PBS, and blocked with 3% BSA for 1 h at room temperature. The primary antibodies against monoclonal KLK2 (M03, clone 3C5, 1:100, Abnova, Taiwan), Ki‐67 (AB9260 Merck, 1:50, Sigma‐Aldrich), AR (clone D611, 5153, 1:500, Cell Signaling Technology), and PSMA/FOLH1/NAALADase I (MAB4234, 1:100, R&D Systems) were added to perform indirect immunostaining. Following incubation overnight at 4°C, the cells were incubated for 1 h at room temperature with the secondary antibodies anti‐mouse FITC (INT‐FP‐SA4000, 1:700, Chemie, Brunschwig) and anti‐rabbit Cy3 (C236, 1:700, Sigma). Counterstaining was accomplished by adding 4′,6‐diamidino‐2‐phenylindole (DAPI) (Sigma, 1:200). Staining was evaluated by a Leica Thunder Imager DMI8 (Leica Microsystems CMS GmbH, Germany).

### 2.7. Reverse Transcription and Quantitative PCR Analysis

Total RNA isolation and purification of treated and nontreated cells were performed with the ReliaPrep RNA Cell Miniprep System (Z6011, Promega, Switzerland) according to the manufacturer’s protocol. A total of 1000 ng of RNA was reverse‐transcribed using the high‐capacity cDNA Reverse Transcription Kit (4368814, Applied Biosystems). Quantitative PCR with TaqMan Fast Advanced Master Mix (4444963, ThermoFisher) of 40 ng cDNA per reaction in a total volume of 10 uL was carried out using the following TagMan primers: KLK2 (Hs00428383_m1), KLK4 (Hs00191772_m1), KLK14 (Hs00983577_m1), PSA (Hs02576345_m1), PSMA (Hs00379515_m1), AR (Hs00171172_m1). The results were normalized to GAPDH (4333764T, TaqMan, ThermoFisher). A QuantStudio 5 (Applied Biosystems) Real‐Time PCR system was used for measurement.

### 2.8. Simple Western (WES) Immunoblotting

Conditioned media were collected and concentrated using Amicon Ultra‐0.5 centrifugal filters (UFC5003, Merck Millipore) at 4°C. Cells were trypsinized and washed with PBS. Cell lysis was performed with a modified lysis buffer supplemented with a protease inhibitor cocktail (Sigma‐Aldrich, Switzerland). After centrifugation at 14′000 × g for 20 min at 4°C, the supernatants were collected for further analysis. Total protein amounts of both supernatant and cell lysates were measured with a BCA Protein Assay Kit (ThermoFisher Scientific, Lausanne, Switzerland). Automated western blot quantitative analysis (ProteinSimple WES, Germany) was performed under reducing conditions according to the manufacturer’s instructions to assess protein expression. Primary antibodies included: mouse anti‐KLK2 (16C6‐2D11‐3B10, 1:50, MedDiscovery, Epalinges, Switzerland), rabbit anti‐AR (5153, 1:100, Cell Signaling Technology), rabbit anti‐PSMA/FOLH1 (MAB4234, 1:25, R&D Systems), rabbit anti‐PSA (5365, 1:100, Cell Signaling Technology), and mouse anti‐GAPDH (1:100, Novus Biologicals Europe) as an internal control. Total protein amounts of 1–2.5 mg/mL were used to load a 12–230 kDa cartridge kit. Data were analyzed using Compass Software v. 5.0.1.

Experiments were not blinded, as data collection and analysis were based on objective, quantitative measurements obtained through standardized and automated procedures such as qPCR, automated western blotting, and flow cytometry. Therefore, blinding was not required for this type of experiment.

### 2.9. Statistical Analysis

GraphPad Prism 8 (GraphPad Software, Inc., La Jolla, CA, version 8) was used for analysis and plots of the data sets. Determination of statistical significance was performed by one‐way analysis of variance (ANOVA) with Bonferroni’s multiple‐comparison post hoc test. *P* values < 0.05 were declared as a significant difference. Data are presented as mean ± standard error of the mean (SEM) of three to five individual experiments each in triplicate if not otherwise stated.

## 3. Results

### 3.1. In Vitro KLK Inhibition Profile of MDPK67b

MDPK67b is a variant of the KLK2 inhibitor ACT6.7 [22] lacking the N‐terminal His‐tag of the original construct. ACT6.7 was developed by phage display screening of ***α*** 1‐antichymotrypsin variants for KLK2 inhibition activity. Besides the previously reported KLK2 inhibition potential of ACT6.7, the molecule and its variant MDPK67b also exhibit strong in vitro inhibition of KLK4, KLK5, and KLK14 proteolytic activity (Table 1). Incubation of KLK4, 5, and 14 with a 20× molar excess of MDPK67b showed complete protease inhibition, while several other KLKs were partially inhibited under the same conditions. The SI values indicate very efficient complex formation of MDPK67b with KLKs 2, 4, and 14 with SI values between 1 and 2 (Table 2). In the following, KLK 2, 4, and 14 will be summarized as KLKs.

### 3.2. Dose‐ and Time‐Dependent Reduction in Cell Viability Upon Pharmacological Inhibition of KLKs With MDPK67b

To evaluate the antitumor effect of KLK inhibition, cell proliferation was assessed in the following PCa cell lines after treatment with different concentrations of MDPK67b at different time points (days 1, 3, 5, and 7): LNCaP (androgen‐sensitive), C4‐2 (AR‐positive cell line growing without androgens but still responding to androgen levels), and DU145 (androgen‐insensitive). To mimic ADT, charcoal‐stripped medium was used for all the experiments. Increasing concentrations of MDPK67b led to a progressive reduction in cell proliferation only in LNCaP, but not in C4‐2 and DU145, which were proliferative until day 7 (Figure 1 and Figure S1). Cell proliferation of LNCaP cells plateaued after day 5 with 0.1 mg/mL MDPK67b (Figure 1(a)). Statistically significant reduction in cell proliferation was observed at day 7 with MDPK67b doses of 0.5 mg/mL to 1 mg/mL. Maximal inhibition was reached at day 5 and day 7 with MDPK67b at 36% and 53%, respectively (Figure 1 and Fig. S1A). In contrast, MDPK67b had no impact on the cell proliferation of the androgen‐independent cell line C4‐2 (Figure 1(b)). Similarly, MDPK67b failed to exert inhibitory effects on DU145, a PCa cell line lacking KLK2 expression (Fig. S1B).

Figure 1Dose‐dependent effect on cell viability in response to MDPK67b treatment. Cells were treated every alternate day with vehicle control and increasing concentrations of MDPK67b (0.1, 0.5, 0.75, and 1 mg/mL) using RPMI without phenol red supplemented with 5% heat‐inactivated charcoal‐stripped FBS. Cell viability of (a) LNCaP and (b) C4‐2 cell lines was measured by CellTiter‐Glo luminescent assay on days 1, 3, 5, and 7. Data are shown as mean ± standard error of the mean (SEM) of three independent experiments in triplicate. Asterisks indicate statistically significant differences (^∗^ *p* ≤ 0.05, ^∗∗^ *p* ≤ 0.01) of treated cells compared to the corresponding vehicle control concentration. Only vehicle control with a concentration of 0.75 mg/mL is illustrated.

**Figure:**
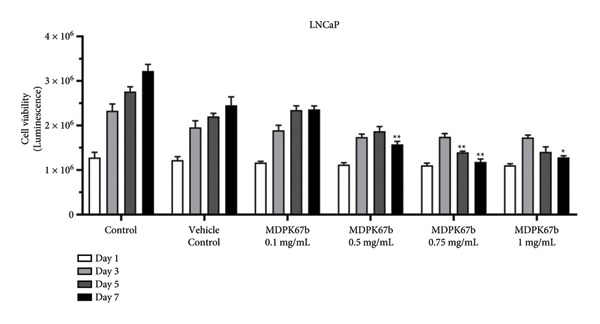
(a)

**Figure:**
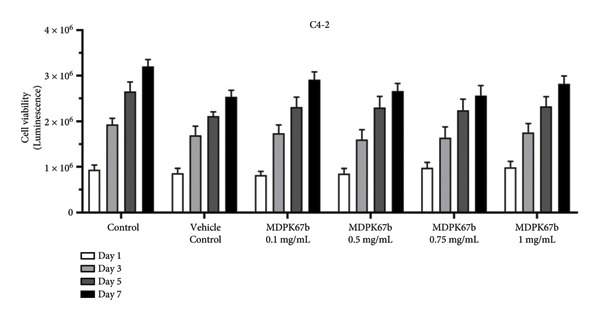
(b)

Taken together, MDPK67b led to a decrease in cell proliferation in a dose‐ and time‐dependent manner. All subsequent experiments were performed with an MDPK67b concentration of 0.75 mg/mL over 5 days.

### 3.3. Induction of Cell Death After MDPK67b Treatment in LNCaP and C4‐2 Cell Lines

To examine the extent of cell death after MDPK67b treatment, apoptosis was evaluated using Annexin V and PI staining by FACS (Figure 2). After treatment with 0.75 mg/mL MDPK67b, an increase in Annexin V expression was observed in LNCaP and C4‐2 cells (Figure 2(a)). In LNCaP, the increase in percentage of positive Annexin V cells was statistically significant (26.51 ± 3.81, *p* < 0.034) (Figure 2(b)). LNCaP cells also showed a significant increase in PI staining after treatment (53.6 ± 4.3, *p* < 0.041) (Figures 2(c), 2(d)). In contrast, DU145 cells did not show an increase in Annexin V or PI expression. The effect of MDPK67b on cell proliferation was quantified by immunostaining of the proliferation marker Ki‐67 (Figure 2(e)). MDPK67b treatment reduced the proportion of Ki‐67–positive cells in LNCaP and C4‐2, but not in DU145 cells.

Figure 2Assessment of cell death by flow cytometry and immunocytochemistry. After a 5‐day incubation period, nontreated and treated cells with 0.75 mg/mL MDPK67b were analyzed for apoptosis (Annexin V) and necrosis (propidium iodide) by flow cytometry. (a) Representative histograms showing Annexin V expression of untreated control (light gray area), vehicle control (black line) and treated cells (dotted line) and (b) the corresponding plots of LNCaP, C4‐2, and DU145 cell lines. Likewise, the histograms and statistical analysis of PI in all the cell lines of control, vehicle control and 0.75 mg/mL MDPK67b (c) and (d). Plots are presented as percentages of positive cells of three independent experiments. Asterisks indicate statistically significant differences (^∗^ *p* ≤ 0.05, ^∗∗^ *p* ≤ 0.01) compared to vehicle control. (e) LNCaP, C4‐2, and DU145 cells were stained with the primary antibody Ki‐67 to illustrate cell proliferation for the different conditions. Counterstaining was performed by DAPI (4′,6‐diamidino‐2‐phenylindole). Scale bars: 50 **μ**m.

**Figure:**
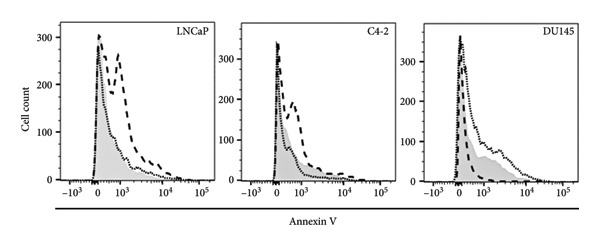
(a)

**Figure:**
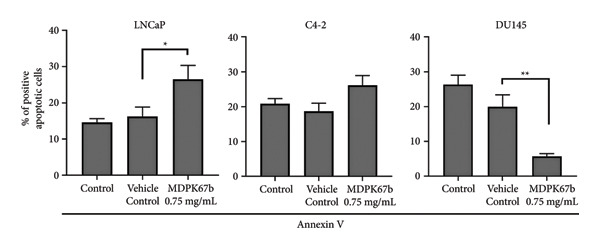
(b)

**Figure:**
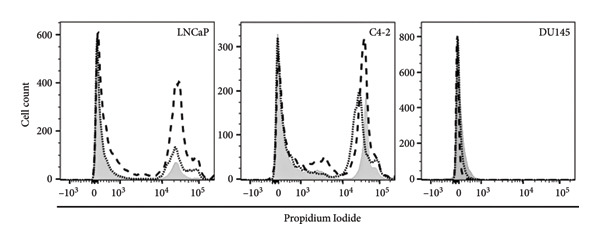
(c)

**Figure:**
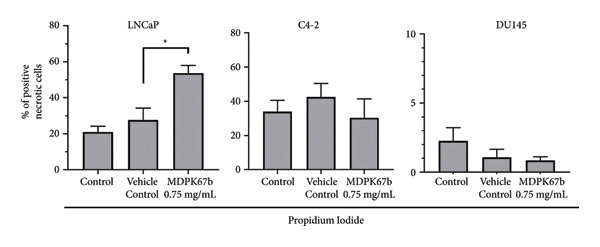
(d)

**Figure:**
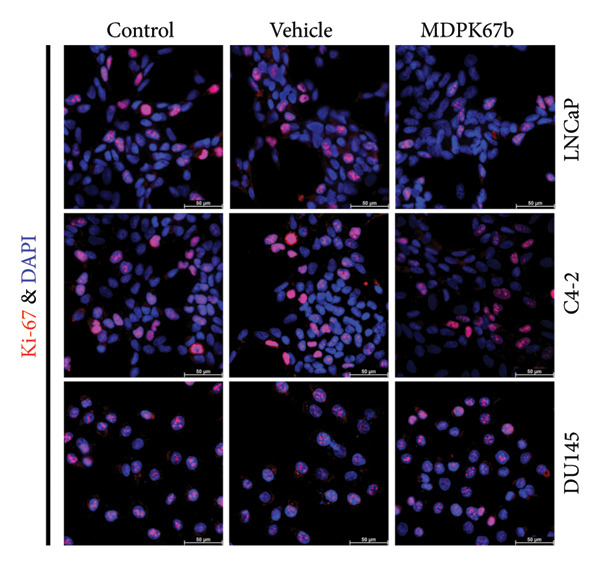
(e)

### 3.4. Downregulation of AR and PSA After KLK Inhibition With MDPK67b Treatment

**Figure 3:**
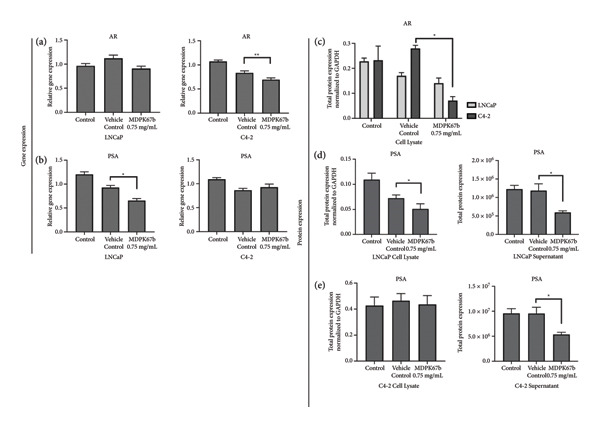
Gene and total protein expression analysis after MDPK67b treatment. Cells were treated as mentioned previously and collected for RNA isolation and cDNA synthesis. mRNA levels were quantified by real‐time PCR using the primers for (a) AR and (b) PSA in LNCaP and C4‐2. Data represent mRNA mean ± SEM from five independent experiments performed in triplicate normalized to GAPDH. Asterisks indicate statistically significant differences (^∗^ *p* ≤ 0.05, ^∗∗^ *p* ≤ 0.01) compared to vehicle control. For protein determination, cells were treated with 0.75 mg/mL MDPK67b for 5 days. Afterward, cells were lysed, and protein was measured by the BCA protein assay kit. Protein samples (1–2.5 mg/mL) were loaded into 12–230 kDa cartridges. The following proteins were quantified: (c) AR cell lysate, (d) PSA in LNCaP cell lysate and supernatant, and (e) PSA in C4‐2 cell lysate and supernatant. Data represent mean ± SEM of three to five experiments each normalized to GAPDH and compared to vehicle control. Asterisks indicate statistically significant differences (^∗^ *p* < 0.05) compared to vehicle control.

It has been proposed that KLK2 has an AR‐independent function and activates PSA via a proteolytic mechanism in the secreted fluid. Therefore, we examined AR and PSA expressions at the gene and protein levels (Figure 3). MDPK67 reduced AR gene expression in C4‐2 cells and decreased PSA levels in LNCaP cells (0.677 ± 0.087, *p* < 0.037) (Figures 3(a), and 3(b)). Expression of KLK4 was slightly increased after treatment in LNCaP cells but reduced in C4‐2 and DU145. None of the changes were statistically significant (Fig. S2). On the other hand, gene expression of KLK14 was increased in all the cell lines after treatment with MDPK67b but only statistically significant in C4‐2 (−0.863 ± −0.321, *p* < 0.0002) (Fig. S2).

The inhibitory effect of MDPK67b on the expression of AR was confirmed in both LNCaP and C4‐2 cells on a protein level, in the latter more pronounced (0.071 ± 0.016, *p* < 0.022) (Figure 3(c)). PSA in cell lysate of treated LNCaP and C4‐2 only showed a significant reduction in LNCaP (0.677 ± 0.087, *p* < 0.037) when compared to vehicle control (Figures 3(d), 3(e)). On the other hand, in the supernatant of both treated LNCaP (595,340 ± 42,687, *p* < 0.02) and C4‐2 (5370282 ± 428,409, *p* < 0.032), significant reductions of PSA levels were detected (Figures 3(d), 3(e)). No KLK2, AR, or PSA expression was detected on a gene or protein level in DU145 (data not shown).

Immunostaining showed a very pronounced downregulation of both KLK2 and AR in LNCaP (Figure 4(a)) and C4‐2 cells (Figure 4(b)). Distribution of KLK2 in the cell is mainly detected in the cytoplasm.

Figure 4Immunohistochemical staining. Representative immunofluorescent staining upon treatment with MDPK67b for 5 days in (a) LNCaP and (b) C4‐2 cells. Images were obtained with a Leica Thunder Imager DMI8 at 63× magnification field. Primary antibodies for staining included KLK2 and AR. Counterstaining was performed by DAPI (4′,6‐diamidino‐2‐phenylindole, 1:200). Scale bars: 50 **μ**m.

**Figure:**
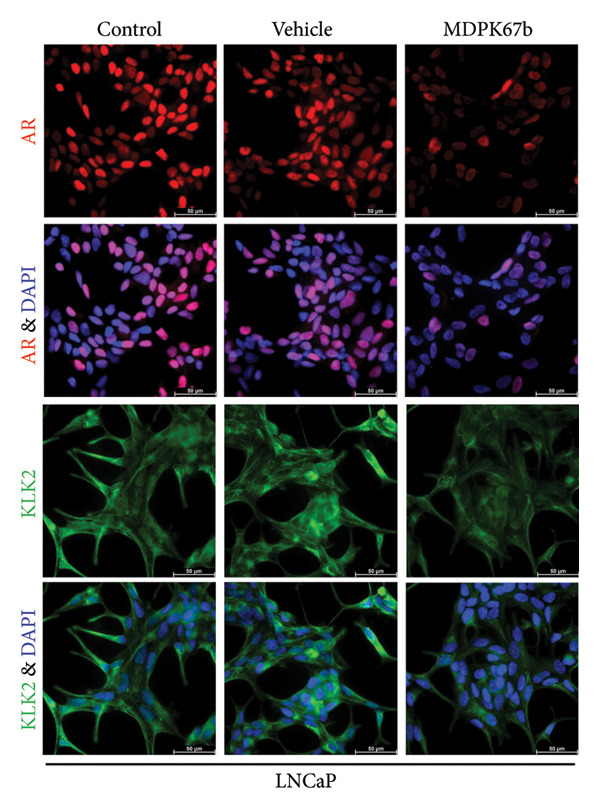
(a)

**Figure:**
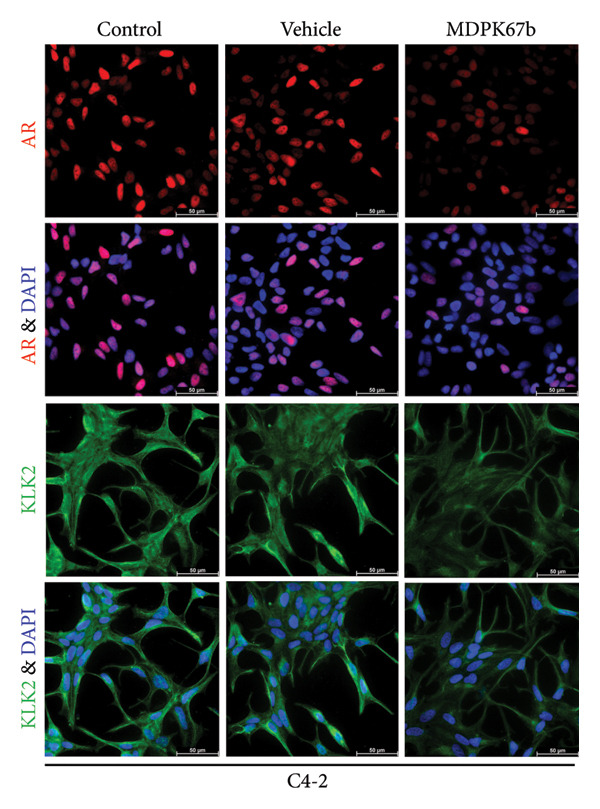
(b)

Taken together, MDPK67b affects both the expression of AR and PSA at the gene and protein levels.

### 3.5. Upregulation of PSMA Upon KLK Inhibition

Next, we assessed the relation of AR downregulation through MDPK67b on the expression of PSMA (Figure 5). Inhibition of KLKs significantly increased PSMA gene expression in both LNCaP (1.34 ± 0.12, *p* < 0.017) and C4‐2 (1.51 ± 0.2, *p* < 0.004) cell lines compared to vehicle control as quantified by qPCR (Figure 5(a)). Equivalent results were observed for protein expression where a highly significant increase in PSMA expression in LNCaP (1.25 ± 0.05, *p* < 0.001) and C4‐2 (1.26 ± 0.02, *p* < 0.007) was revealed (Figure 5(b)). The results were confirmed by immunostaining, illustrating the increased PSMA expression in LNCaP and C4‐2 after treatment with MDPK67b (Figures 5(c) and 5(d)).

Figure 5Effect of KLK inhibition on PSMA expression. Gene (a) and protein (b) expression in LNCaP and C4‐2 cell lines was performed as mentioned in the previous figures. Data represent mRNA mean ± SEM of five experiments in triplicate and protein expression mean ± SEM of three to five experiments each normalized to GAPDH and compared to vehicle control. Asterisks indicate statistically significant differences (^∗^ *p* ≤ 0.05, ^∗∗^ *p* ≤ 0.01) compared to vehicle control. (c) Images of the conformational immunostaining of the primary antibody PSMA after treatment with MDPK67b for 5 days in LNCaP and C4‐2 were taken with a Leica Thunder Imager DMI8 at 63× magnification field. Counterstaining by DAPI (4′,6‐diamidino‐2‐phenylindole, 1:200). Scale bars: 50 **μ**m. (d) Graph indicates quantitative measurements of fluorescent intensity using ImageJ software. ^∗∗∗^ *p* ≤ 0.001 and ^∗∗∗∗^ *p* ≤ 0.0001 indicate statistically significant differences compared to vehicle control (*n* = 10 per condition of 63× microscopic field images).

**Figure:**
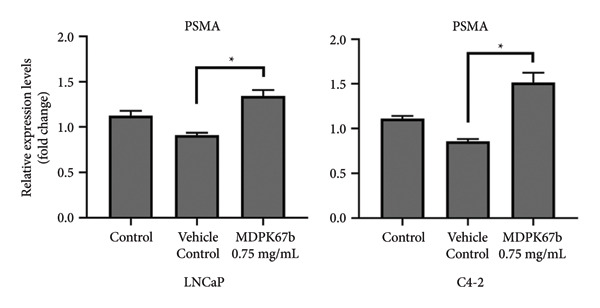
(a)

**Figure:**
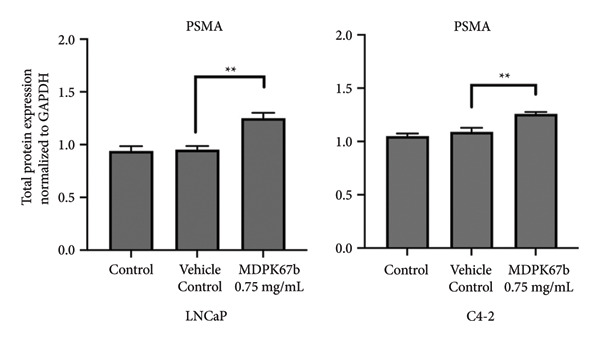
(b)

**Figure:**
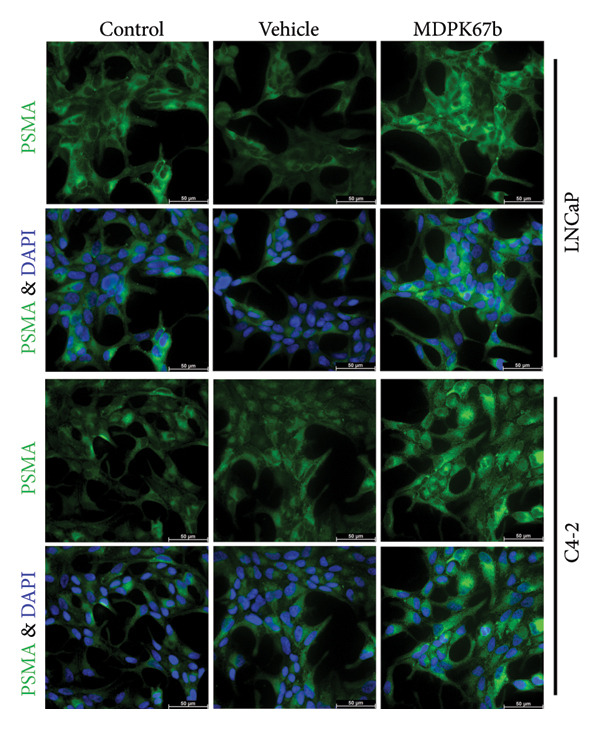
(c)

**Figure:**
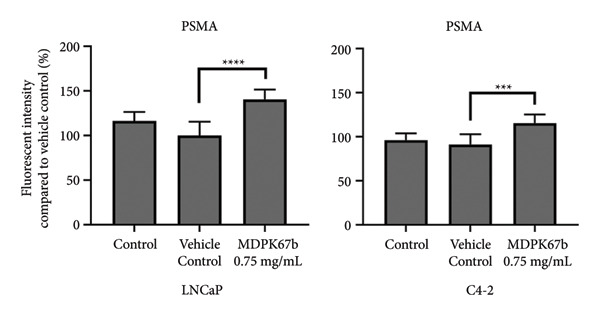
(d)

## 4. Discussion

In this study, we demonstrate the pharmacological antitumor effect of MDPK67b, a recombinant protease inhibitor targeting human kallikrein‐related peptidase 2, 4, and 14, in PCa cell lines in vitro. The effect is seen mainly in LNCaP, but not in C4‐2 and DU145. In addition, we show that blockage of KLK2 protease activity and of prostate cell–derived trypsin‐like KLKs inhibits the AR signaling in a retrograde manner in both the androgen‐dependent (LNCaP) and ‐independent cell line (C4‐2). This confirms that MDPK67b influences the expression of PSA and PSMA. These results confirm that the PCa‐specific KLK2 is a potential therapeutic and also diagnostic target in advanced and CRPCa.

Darson et al. showed that the trypsin‐like serine protease KLK2 is mainly expressed in the prostate gland but increasingly in advanced and metastasized PCa tissue in a study from 1999 [10]. Several mechanisms by which KLK2 stimulates cell proliferation and invasion are described. Proteolytic degradation of insulin‐like growth factor binding protein‐3 (IGFBP‐3) by KLK2 leads to complex dissociation of IGFBP‐3 and IGF, thereby stimulating proliferation in vitro [25]. Protease‐activated receptor 2 (PAR2), a G protein‐coupled receptor involved in PCa development and progression, is activated in DU145 PCa cells by recombinant KLK2, resulting in a stimulation of the ERK1/2 signaling pathway [13]. In addition, KLK2 is capable of activating the zymogen form of uPA, a crucial extracellular protease correlated with cancer invasion and metastasis [26].

Not only KLK2, but also KLK4 and KLK14influence key signaling pathways in PCa tumorigenesis by their enzymatic activities, illustrated by Cereda et al. [27]. KLK4 knockdown resulted in a significant reduction of PCa cell growth in vitro and in vivo by interaction with promyelocytic leukemia zinc finger (PLZF) and resulting regulation of the mTORC1 signaling pathway [28]. In addition, interaction of KLK4 with PLZF hinders the AR activation. This emphasizes the role and crosstalk of the KLKs with AR signaling and major proliferation pathways. Also, simultaneous inhibition of additional proteases might be required for maximizing the in vitro efficiency. Therefore, the use of protease inhibitors with a broader substrate specificity reacting with different trypsin‐like KLKs might increase the potential of the inhibitor when compared to a sole KLK2‐specific molecule.

Through blockage of KLKs in the secreted cell culture media, we confirmed the reduction in cell viability in LNCaP cells and showed a decrease of Ki‐67, a proliferation marker with prognostic relevance in PCa [29]. Moreover, inhibition of KLKs induced cell death detected by Annexin V and PI in FACS. The androgen‐sensitive but independent cell line C4‐2, on the other hand, did not show reduced cell proliferation, emphasizing a possible bypass mechanism, where the cells can hold up the proliferative phase even though a slight increase in Annexin V was detected.

Cell survival and proliferation in PCa is regulated through AR and its target genes. In CRPCa, AR signaling is often found to remain activated, which explains the focus in current therapeutic approaches on AR inhibition. Second‐line AR pathway inhibitors like Enzalutamide, an AR‐inhibitor, showed an increase in survival of treated patients [30], but a persistent effect fails to appear, and development of drug resistance is currently a major clinical problem [31]. The mechanisms of CRPCa are still poorly understood. Bypass of the AR and activation of downstream targets seem to play an important role. Arora et al. demonstrated a mechanism in enzalutamide‐resistant disease where an upregulation of the glucocorticoid receptor can bypass AR blockage and induce expression of KLK2, but not PSA or PSMA [32]. This setting makes KLK2 an even more interesting target for therapy in CRPCa and supports the AR‐independent approach. Additionally, Shang et al. demonstrated that KLK2 can function as an AR modulator through transactivation of the AR coregulator ARA70 and thereby modulate cell growth in CRPCa [15]. In this study, we show that pharmacological inhibition of secreted KLKs with MDPK67b has a retrograde effect on the AR and thereby triggers an antitumor effect in vitro, corroborating Shang’s findings. Even in the AR‐independent but AR‐sensitive cell line C4‐2, a reduction of AR expression was achieved by MDPK67b treatment.

Another relevant downstream target gene of AR is PSMA. Like KLK2, PSMA expression is higher in primary PCa as compared to benign prostate tissue, but even higher in metastatic PCa [33, 34]. In recent years, PSMA has become an important theranostic tool with relevance in clinical decision making [35]. PSMA PET‐CT scans are regularly used to assess disease recurrence or progression [2]. Radiolabeling of PSMA with 177Lu has become a further treatment option for metastatic CRPCa [36, 37]. It has been shown that the AR synthesis blocking by Abiraterone increases PSMA expression in vitro [38]. Likewise, short‐term treatment with Enzalutamide, an AR signaling cascade inhibitor, leads to re‐expression of the PSMA surface levels [39]. Our study confirms significant upregulation of PSMA by AR downregulation through blockage of KLKs with a 5‐day course treatment of MDPK67b in vitro in LNCaP and C4‐2 cell lines. This was demonstrated at both gene and protein levels. This effect of MDPK67b to boost PSMA expression needs to be further evaluated and may be important in clinical studies using PSMA in diagnostics and therapy. Exploring potential combined therapeutic strategies, for instance, integrating MDPK67b with the established AR pathway inhibitors or PSMA‐targeted approaches, could further enhance its translational relevance and strengthen the future therapeutic outlook.

However, there is a general limitation using PSMA as a target: it has a high expression in other organs and in radiosensitive tissues. Therefore, it is not prostate tissue–specific, which limits diagnostic accuracy and therefore shows off‐target accumulation in radiolabeled therapy of PCa [40]. These facts favor KLK2, as a complementary or alternative theranostic agent. Timmermand et al. already successfully achieved delivering therapeutic absorbed doses of 177Lu labeled hu11B6, a specific antibody targeting KLK2, in PCa xenografts [41]. Tumor volumes and growth rates were significantly decreased after treatment, and Ki‐67 expression was negatively correlated with the activity uptake of 177Lu‐hu11B6. The Memorial Sloan Kettering Cancer Center (MSKCC) is currently recruiting patients in a Phase I Study of JNJ‐69086420 for advanced PCa. Promising results have been described in a phase 1 trial using transforming growth factor–insensitive, PSMA CAR T cells in CRPCa [42].

Recent studies emphasize the relevance of serum KLK2 in the diagnostic and prognostic workup of PCa patients [43]. Especially with tests like the Stockholm3 (STHLM3) test [44] and the improvement of PCa risk stratification, we face the development of personalized treatments according to cancer profiles. As we demonstrated, treatment of C4‐2 cells with MDPK67b affected AR, PSA, and PSMA expression at the gene and protein levels; however, cell proliferation was not influenced. In contrast, a pronounced anticancer effect was observed in LNCaP cells. In CRPCa, the therapeutic goal is to suppress tumor proliferation, which we were unable to demonstrate in our study. Therefore, the therapeutic impact of targeting KLK2 may be restricted. The heterogeneity of CRPCa suggests that inhibition of KLK2 alone may be insufficient in AR‐negative or highly dedifferentiated tumors. Although the current findings demonstrate that MDPK67b suppresses proliferation in AR‐dependent LNCaP cells, further studies in additional AR‐dependent and CRPCa cell lines, along with in vivo validation, are necessary to fully evaluate the therapeutic potential of kallikrein inhibition. Differences in protease expression patterns, bioavailability, and pharmacodynamic behavior may influence the overall response and should be carefully assessed in subsequent experiments. These limitations should be taken into account when considering the translational relevance of KLK2‐directed therapies.

## 5. Conclusion

In conclusion, MDPK67b represents a promising therapy option in CRPCa. Inhibition of KLKs leads to a significant reduction in cell proliferation in LNCaP. However, AR‐dependent and independent drug resistance point to the complex biology of CRPCa. In the C4‐2 cells, the effect of MDPK67b on the AR and its downstream target genes PSA and PSMA was achieved without influencing proliferation. In light of the MDPK67b‐mediated enhancement of PSMA, the inhibition of KLK function may optimize PSMA targeted therapy. The effect of MDPK67b presented in our study is worth pursuing with the aim for clinical implementation.

**Nomenclature**
AR: androgen receptor
ADT: androgen deprivation therapy
CR: castration‐resistant
CRPCa: castration‐resistant prostate cancer
DAPI: 4′,6‐diamidino‐2‐phenylindole
FBS: fetal bovine serum
IGFBP: insulin‐like growth factor binding proteins
KLK: kallikrein‐related peptidase
PAR: protease‐activated receptors
PBS: phosphate buffered saline
PCa: prostate cancer
PI: propidium iodide
PLZF: promyelotic leukemia zinc finger
PSA: prostate‐specific antigen
PSMA: prostate‐specific membrane antigen
uPA: urokinase‐type plasminogen activator

## Conflicts of Interest

Christoph Kündig is a salaried employee and minority shareholder of MedDiscovery SA without a competing interest.

## Author Contributions

Fabienne Lehner: conceptualization, methodology, validation, formal analysis, investigation, writing–original draft, and visualization. Christoph Kündig: conceptualization, methodology, validation, formal analysis, resources, writing–review and editing. Christopher Millan: methodology, validation, investigation, writing–review and Editing. Souzan Salemi: conceptualization, methodology, validation, formal analysis, investigation, resources, writing–review and editing, and visualization. Daniel Eberli: conceptualization, methodology, resources, and writing–review and editing.

## Funding

This work was supported by the Theodor and Ida Herzog‐Egli Stiftung, Switzerland, and the Julius‐Müller‐Stiftung, Switzerland. Open access publishing facilitated by Universitat Zurich, as part of the Wiley ‐ Universitat Zurich agreement via the Consortium of Swiss Academic Libraries.

## Supporting Information

Additional supporting information can be found online in the Supporting Information section.

## Supporting information


**Supporting Information 1** Figure S1: (A) Dose‐dependent effect on cell viability in LNCaP cells in response to MDPK67b treatment. For a conclusive evaluation, cell viability of LNCaP cells treated with 0.75 mg/mL MDPK67b over 7 days was compared to vehicle control (0.75 ng/mL) as a percental change. Plot represents percentages compared to vehicle control. (B) Cell viability of DU145 cells in response to the different doses of MDPK67b treatment. Cell viability was measured by CellTiter‐Glo® luminescent assay on days 1, 3, 5, and 7. Data are shown as mean ± SEM of three independent experiments in triplicate. Compared to the corresponding vehicle control concentration, there is no significant change. Only vehicle control with a concentration of 0.75mg/mL is illustrated.

**Supporting Information 2** Figure S2: Gene expression analysis of KLKs after MDPK67b treatment. Cells were treated as mentioned previously and collected for RNA isolation and cDNA production. mRNA levels were quantified by real‐time PCR of the following primers in LNCaP and C4‐2: KLK2, KLK4, and KLK14. Bar graphs show fold change of gene expression compared to vehicle control. Data represent mRNA mean ± SEM of five experiments in triplicate each normalized to GAPDH. Asterisks indicate statistically significant differences (^∗^ *p* ≤ 0.05) compared to vehicle control.

## Data Availability

The data that support the findings of this study are available from the corresponding author upon reasonable request.
