# Supplementary material for: Inhibition of Growth and Induction of Apoptosis of Human Prostate Cancer Cells by Enzymatic Blockage of Kallikreins
Source: Prostate Cancer. 2026 Jan 11;2026:7871208. doi: 10.1155/proc/7871208 (PMC12791160; doi:10.1155/proc/7871208)
Supplement: Supplementary file 1 — Supporting Information 1 Figure S1: (A) Dose‐dependent effect on cell viability in LNCaP cells in response to MDPK67b treatment. For a conclusive evaluation, cell viability of LNCaP cells treated with 0.75 mg/mL MDPK67b over 7 days was compared to vehicle control (0.75 ng/mL) as a percental change. Plot represents percentages compared to vehicle control. (B) Cell viability of DU145 cells in response to the different doses of MDPK67b treatment. Cell viability was measured by CellTiter‐Glo® luminescent assay on days 1, 3, 5, and 7. Data are shown as mean ± SEM of three independent experiments in triplicate. Compared to the corresponding vehicle control concentration, there is no significant change. Only vehicle control with a concentration of 0.75mg/mL is illustrated. [file PROC-2026-7871208-s002.pdf]

Supplementary Figure 1

A

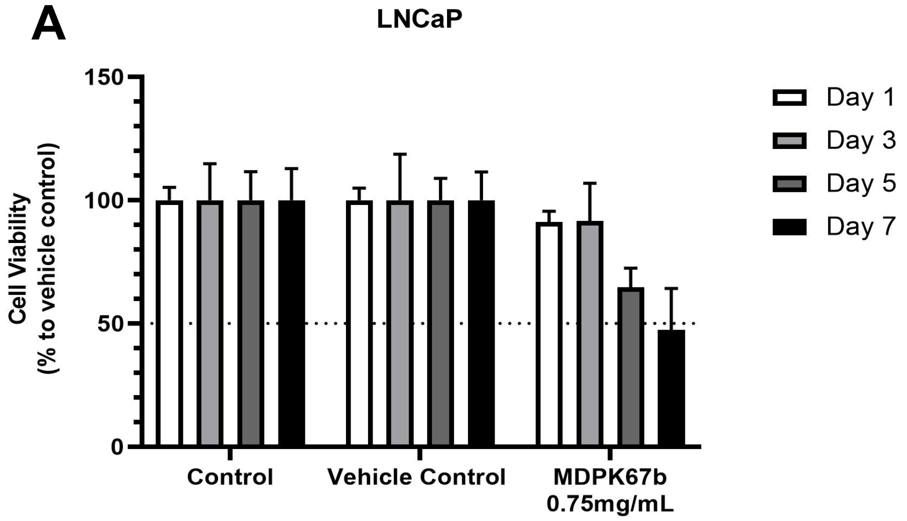

B

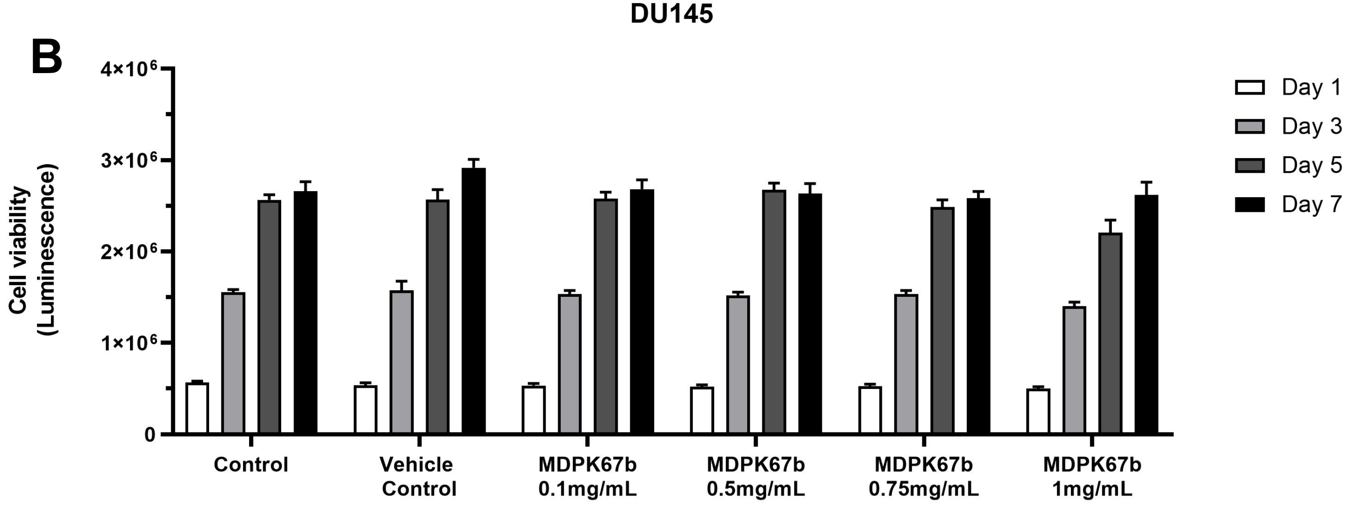

(**A**) **Dose-dependent effect on cell viability in LNCaP cells in response to MDPK67b treatment.** For a conclusive evaluation, cell viability of LNCaP cells treated with 0.75 mg/mL MDPK67b over 7 days was compared to vehicle control (0.75 ng/mL) as a percental change. Plot represents percentages compared to vehicle control.

(**B**) **Cell viability of DU145 cells in response to the different doses of MDPK67b treatment.** Cell viability was measured by CellTiter-Glo® luminescent assay on day 1, 3, 5 and 7. Data are shown as mean ± standard error of the mean (SEM) of three independent experiments in triplicates. Compared to the corresponding vehicle control concentration there is no significant change. Only vehicle control with a concentration of 0.75mg/mL is illustrated.
