# Supplementary material for: Inhibition of Growth and Induction of Apoptosis of Human Prostate Cancer Cells by Enzymatic Blockage of Kallikreins
Source: Prostate Cancer. 2026 Jan 11;2026:7871208. doi: 10.1155/proc/7871208 (PMC12791160; doi:10.1155/proc/7871208)
Supplement: Supplementary file 2 — Supporting Information 2 Figure S2: Gene expression analysis of KLKs after MDPK67b treatment. Cells were treated as mentioned previously and collected for RNA isolation and cDNA production. mRNA levels were quantified by real‐time PCR of the following primers in LNCaP and C4‐2: KLK2, KLK4, and KLK14. Bar graphs show fold change of gene expression compared to vehicle control. Data represent mRNA mean ± SEM of five experiments in triplicate each normalized to GAPDH. Asterisks indicate statistically significant differences (∗ p ≤ 0.05) compared to vehicle control. [file PROC-2026-7871208-s001.pdf]

# Supplementary Figure 2

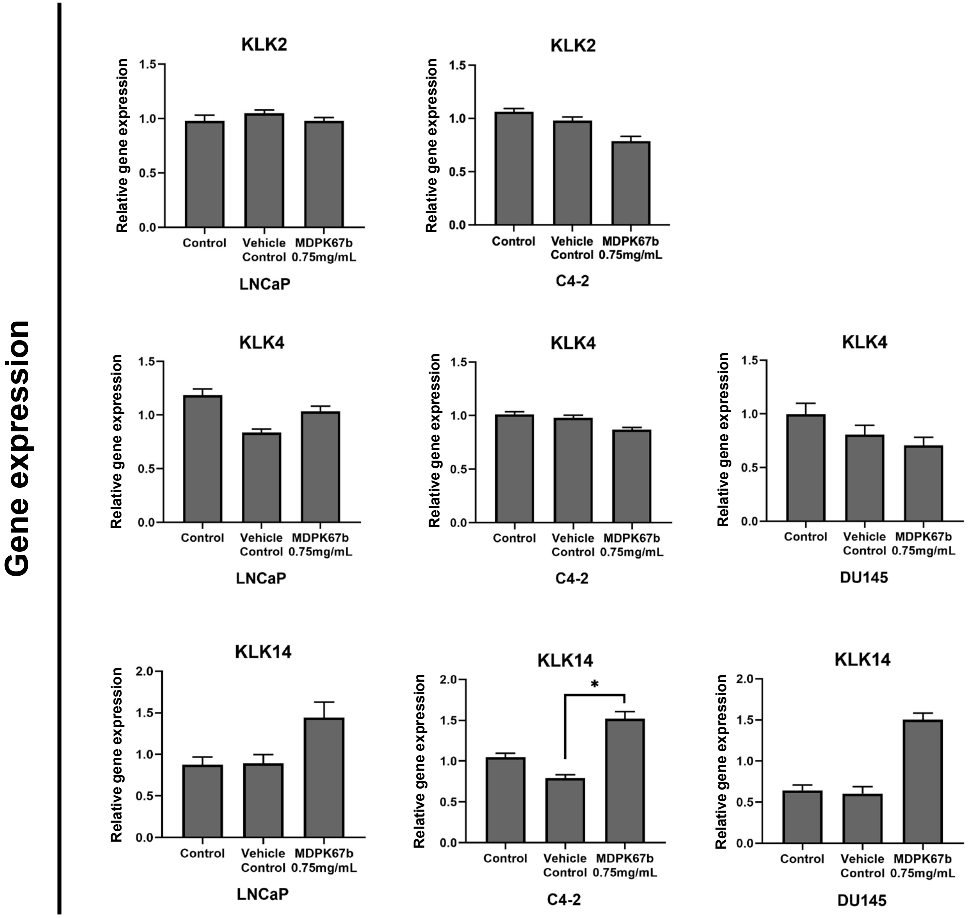


**Gene expression analysis of KLKs after MDPK67b treatment.** Cells were treated as mentioned previously and collected for RNA isolation and cDNA production. mRNA levels were quantified by real-time PCR of the following primers in LNCaP and C4-2: KLK2, KLK4 and KLK14. Bar graphs show fold change of gene expression compared to vehicle control. Data represent mRNA mean ± SEM of five experiments in triplicates each normalized to GAPDH. Asterisks indicate statistically significant differences (*p≤0.05) compared to vehicle control.
